# Huoxiang Zhengqi dropping pills alleviate exertional heat stroke–induced multiple organ injury through sustaining intestinal homeostasis via regulating MAPK/NF-κB pathway and gut microbiota in rats

**DOI:** 10.3389/fphar.2024.1534713

**Published:** 2025-01-07

**Authors:** Lei Li, Jun Ma, Zeshi Li, Juelin Chen, Jiawei Zhou, Yawei Wang, Yankun Pei, Yitong Gong, Jianyao You, Yangyang Cao, Man Wang, Jikuai Chen, Wenjun Chang, Weiyi Ma, Hanyu Zhu, Chuhan Xiang, Shuogui Xu, Qing Song

**Affiliations:** ^1^ Heat Stroke Treatment and Research Center of Chinese PLA General Hospital, Sanya, China; ^2^ Department of Emergency, Changhai Hospital, Naval Medical University, Shanghai, China; ^3^ Department of Emergency, The Second Naval Hospital of Southern Theater Command of PLA, Sanya, China; ^4^ Department of Critical Care Medicine, Hainan Hospital, Chinese PLA General Hospital, Sanya, China; ^5^ Department of Rehabilitation and Traditional Chinese Medicine, Changhai Hospital, Naval Medical University, Shanghai, China; ^6^ Department of Health Toxicology, Faculty of Naval Medicine, Naval Medical University, Shanghai, China; ^7^ Faculty of Naval Medicine, Naval Medical University, Shanghai, China; ^8^ Department of Emergency, First Medical Center of Chinese PLA General Hospital, Beijing, China; ^9^ National Clinical Research Center for Kidney Diseases, Beijing Key Laboratory of Kidney Diseases Research, Department of Nephrology, First Medical Center of Chinese PLA General Hospital, Beijing, China; ^10^ Department of Cardiology, The Affiliated Huaihai Hospital of Xuzhou Medical University, Xuzhou, Jiangsu, China

**Keywords:** exertional heat stroke, heat stress, heat tolerance, dietary supplement, intestinal barrier, gut microbiota

## Abstract

Exertional heat stroke (EHS) is a life-threatening condition characterized by hyperthermia and multi-organ dysfunction, often associated with intestinal barrier disruption. This study evaluated the protective effects of Huoxiang Zhengqi Dropping Pills (HXZQD) against EHS in a rat model. HXZQD was administered via oral gavage at low, medium, and high doses, followed by EHS induction through exercise under high-temperature and high-humidity conditions. The findings revealed that high-dose HXZQD significantly delayed the onset of EHS, reduced core body temperature elevations, and mitigated multi-organ injury, as evidenced by biochemical markers and histopathological examination. This study showed that HXZQD alleviated EHS-induced intestinal damage by preserving barrier proteins (ZO-1, Occludin, and Ecadherin) and maintaining intestinal barrier integrity. Transmission electron microscopy confirmed the preservation of tight junction structures. Further analysis indicated that HXZQD modulated the MAPK/NF-κB signaling pathways, inhibiting heat stress-induced activation and reducing inflammation. Additionally, HXZQD positively regulated gut microbiota, increasing the proportion of beneficial *Lactococcus* and decreasing harmful *Streptococcus*. These findings suggest that HXZQD maintains intestinal homeostasis during EHS by preserving barrier function and modulating gut microbiota, offering a promising preventive approach for EHS management.

## 1 Introduction

Exertional heat stroke (EHS) is a life-threatening condition caused by intense physical activity in hot environments, characterized by hyperthermia and multi-organ dysfunction (Bouchama et al., 2022). With the intensifying trend of global warming and the increasing frequency of heatwaves worldwide, the incidence of EHS is steadily rising (Raymond et al., 2020; Ahima, 2019). According to China’s expert consensus, EHS, as a subtype of heat stroke, predominantly occurs in healthy young individuals such as soldiers, athletes, firefighters, and outdoor workers. These susceptible population is often required to perform high-intensity activities in hot environments, which are typically unavoidable. Therefore, developing preventive measures specifically for these populations is crucial and can effectively reduce the incidence and progression of heat stroke. Traditional Chinese Medicine (TCM), rooted in a distinctive theoretical framework, boasts a long-standing history and extensive clinical experience in the treatment of heat stroke. Herbal therapies, encompassing traditional herbal formulas, Chinese patent medicines, single Chinese herbs, as well as their extracts or active components, have been utilized and studied for the treatment of heat stroke throughout Chinese history. Our research group has previously conducted a comprehensive summary of various TCM compounds for combating heat stroke (Li et al., 2023). Huoxiang Zhengqi San, a traditional formula documented in the *Taiping Huimin Hejiju Fang* (*Prescriptions of Peaceful Benevolent Dispensary*), is composed of 10 key herbs and has been used for over 900 years (Xiao et al., 2020). Renowned for its ability to induce diaphoresis, clear heat, resolve dampness, and support spleen and stomach function, this formula is available in various forms, including capsules, granules, oral liquids, and pills. A study demonstrates that Huoxiang Zhengqi Dropping Pills (HXZQD) effectively prevent acute intestinal injury caused by heatstroke by enhancing the expression of claudin-3. For EHS-susceptible populations required to work in hot environments, no studies have yet confirmed the protective effects of HXZQD against EHS. Notably, in China, HXZQD is widely regarded as an essential dietary supplement for combating EHS during various high-temperature activities (Li et al., 2022). There is an urgent need for experimental studies to confirm its protective effects and elucidate its underlying mechanisms. In this study, experiments were conducted to validate the protective effects of HXZQD against EHS and to investigate its potential mechanisms.

## 2 Materials and methods

### 2.1 Animals and treatment

**FIGURE 1:**
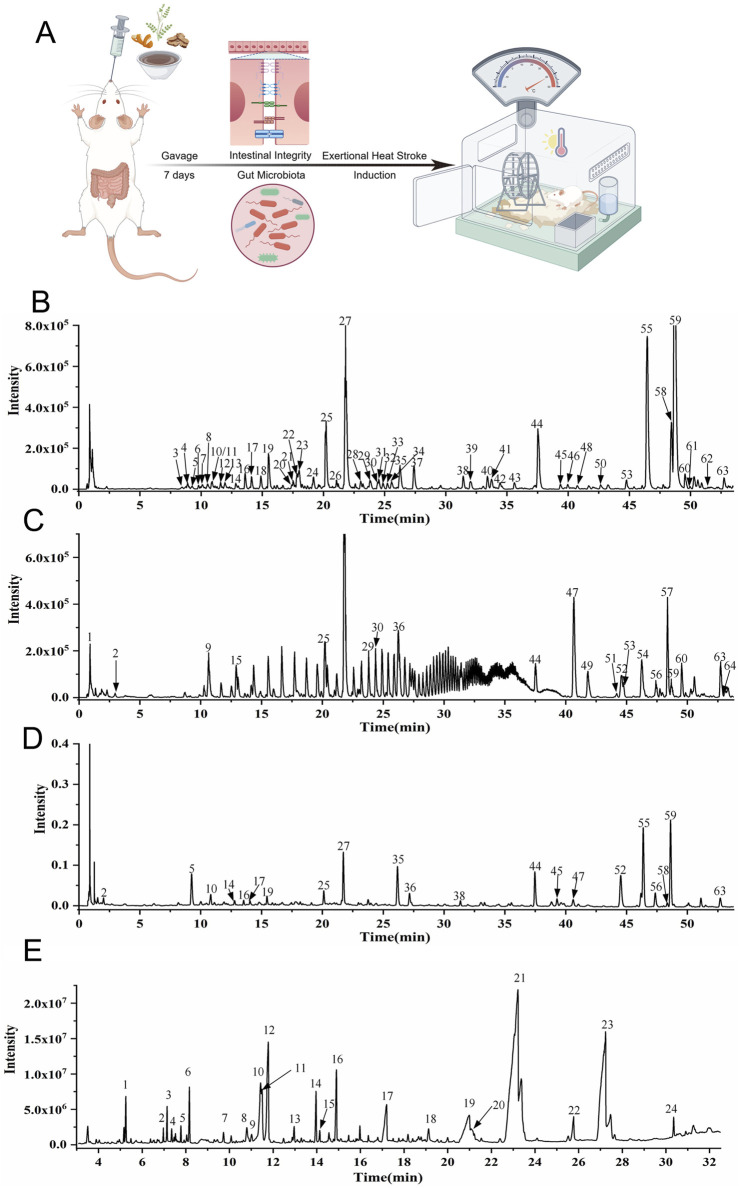
UPLC-Q-TOF-MS and GC-MS analysis of HXZQD and experimental design. **(A)** Rats first underwent a 7-day intragastric administration of HXZQD, followed by EHS induction. **(B)** UPLC-HRMS Base Peak Chromatogram, Negative Ion Mode. **(C)** UPLC-HRMS Base Peak Chromatogram, Positive Ion Mode. **(D)** UPLC UV Chromatogram—UV 254 nm. **(E)** Total Ion Chromatogram.

Male Sprague-Dawley rats (7–8 weeks old, 250–300 g) were obtained from B&K Laboratory Animal Ltd. (Shanghai, China). The rats were housed in the SPF Animal Experiment Center of the Naval Medical University and acclimated for 1 week under 22°C ± 1°C, 50% ± 5% RH, and a 12-h light/dark cycle. All procedures were approved by the Naval Medical University Institutional Animal Ethics Committee (NMUMREC-2021-002) following the NIH Guide for the Care and Use of Laboratory Animals. The sample of HXZQD (National Drug Approval Number: Z20000048, Tasly Pharma Co., Ltd., Tianjin, China) is a commercially purchased product, and a retain sample has been stored in our laboratory for quality control and future reference. The formula of HXZQD consists of several herbs, including Atractylodes Rhizome [Cang Zhu, the rhizome of *Atractylodes lancea (Thunb.) DC.*], dried tangerine peel (Chen Pi, the aged peel of the ripe fruit from *Citrus reticulata Blanco*), Magnolia Bark [Jiang Hou Pu, *Houpoea officinalis (Rehder & E.H. Wilson) N.H. Xia & C.Y. Wu* processed with ginger], Angelica Dahurica [Bai Zhi, *Angelica dahurica (Fisch. ex Hoffm.) Benth. et Hook.*], Poria [Fu Lin, *Wolfiporia cocos (Schw.) Ryv. & Cilbn.*], Areca Peel (Da Fu Pi, the peel of *Areca catechu L., 1753*), Pinellia Rhizome [Sheng Ban Xia, *Pinellia ternate (Thunb.) Makino*], Licorice Extract (Gan Cao Jin Gao, a concentrated preparation derived from the roots of *Glycyrrhiza uralensis Fisch.*), Patchouli Oil [Guang Huo Xiang You, an essential oil extracted from the leaves and stems of *Pogostemon cablin (L.) H.S.Irwin & Barneby*], and Perilla Leaf Oil [Zi Su Ye You, an essential oil extracted from the leaves of *Perilla frutescens (L.) Britton, 1894*]. Suspensions of HXZQD were prepared by mixing the calculated pill weight for each group with 30 mL sterile distilled water in a 50 mL EP tube, then ultrasonicated at 23°C, 100% power for 60 min. Before gavage, the suspensions were shaken well. Based on body weight, the required volume was calculated and administered via gavage using a 2.5 mL syringe. The control and dose groups received gavage daily for 7 days. Finally, based on the intervention concentrations of HXZQD (0.23 g/kg, 0.46 g/kg, and 0.92 g/kg) and the presence or absence of EHS induction, the rats were divided into eight groups: Con, HXL (0.23 g/kg), HXM (0.46 g/kg), HXH (0.92 g/kg), Con + EHS, HXL + EHS (0.23 g/kg), HXM + EHS (0.46 g/kg), and HXH + EHS (0.92 g/kg). The selection of this dosage was primarily based on a study reporting the protective effects of HXZQD against classic heat stroke. We referred to this study to determine the appropriate dosage (Li et al., 2022). After 7 days of gavage treatments, the EHS rats were subjected to heat stress to induce EHS (Figure 1A).

### 2.2 UPLC-Q-TOF-MS and GS-MS identification of major HXZQD metabolites

A 1.0 g sample of HXZQD was added to 10 mL 80% methanol in a 50 mL corked conical flask. The GS solution was solubilized with ultra sound (300 W, 40 kHz) for 30 min. Then the GS solution was centrifuged at 12,000 g for 5 min to obtain supernatant. Ultra-performance liquid chromatography-quadrupole time-of-flight mass spectrometry (UPLC-Q-TOF-MS) analysis was performed on an Agilent 1290 UPLC system (Agilent, United States) in tandem with an AB Sciex Triple TOF 4600^®^ quadrupole-time of flight mass spectrometer (AB SCIEX, United States) equipped with a DuoSpray source (electrospray ionization, ESI). Acquity UPLC^®^ HSS T3 column (2.1 × 100 mm, 1.8 μm; Waters) was used for component separation. The column oven temperature was set at 30°C. The flow rate was 0.3 mL/min. The detection wave length was from 190 to 400 nm. The mobile phase consisted of water with acetonitrile (A) and 0.1% formic acid (B). The gradient elution protocol was as follows: 0–3 min, 97% B; 3–8 min, 97%–90% B; 8–25 min, 90%–75% B; 25–42 min, 75%–55% B; 42–54 min, 55%–25% B; 54–59 min, 25%–5% B; 59–62 min, 5% B; 62–62.1 min 5%–97% B; 62.1–65 min 97% B. Ionization was conducted using an electrospray ionization (ESI) source. Data were collected under both positive and negative ion modes. Mass spectrometry (MS) conditions were as follows: TOF mass range, 50–1,700; ion source gas 50 psi; ion source gas 50 psi; curtain gas, 35 psi; ion spray voltage floating, −4,500/5,000 V; ion source temperature, 500°C; declustering potential, 100 V; MS/MS mass range, 50–1,250; collision energy, ±40 eV; collision energy spread, 20 eV; ion release delay, 30 ms; ion release width 15 ms. Data were collected using an Analyst TF 1.7.1 (AB SCIEX, Foster City, CA, United States) and processed using Peak view 1.2 (AB SCIEX, United States). The main peaks in the fingerprints were identified and authenticated using the Natural Products HR-MS/MS Spectral Library 1.0 software (Shanghai Standard Technology Co., ltd., Shanghai, China). Chemical constituents that were not found in the database were identified using literature reports and the fragmentation patterns in MS. GC-MS analysis was conducted using an Agilent 6890-5977A GC/MSD system (Agilent Technologies) with compound identification performed through comparison with the NIST database. Approximately 5.2 g of the sample of HXZQD was accurately weighed, coating crushed, and dissolved in 20 mL water via ultrasonication (300 W, 40 kHz). The cooled solution was extracted thrice with 20 mL ethyl acetate. Combined extracts were evaporated to dryness, and the residue was dissolved in 2 mL methanol. After centrifugation (12,000 rpm, 5 min), the supernatant was collected for analysis. GC Conditions: Column: HP-5 ms (30 m × 250 μm × 0.25 µm); injection volume: 5 μL; split ratio: 5:1; flow rate: 1.0 mL/min; inlet temperature: 220°C; detector temperature: 280°C. Temperature program: 100°C (initial), ramped at 10°C/min to 165°C (hold 5 min), then to 210°C at 10°C/min (hold 8 min), to 225°C at 5°C/min (hold 2 min), and finally to 280°C at 20°C/min (hold 5 min). MS Conditions: EI ionization; quadrupole temperature: 150°C; ion source temperature: 230°C; scan range: 50–650 Da; electron energy: 70 eV; solvent delay: 3 min; carrier gas: helium; library: NIST11.

### 2.3 Exertional heat stroke protocol and sample collection

The EHS induction protocol was conducted according to our previously reported heat stroke study with improvements (Li et al., 2024a; Li et al., 2024b; Li et al., 2021; He et al., 2020). Rats were subjected to adaptive feeding for 7 days, followed by adaptive treadmill running training. The rats were placed on a Running Wheel Treadmill (KW-ZLP; Nanjing Calvin Biotechnology Co., Ltd.) for exercise. On the first day, the training duration was set to 20 min, increasing by 10 min per day until reaching 60 min on the fifth day. Each training session began at a speed of 5 m/min, with an increment of 1 m/min every 2 min, until reaching 10 m/min. Once the speed reached 10 m/min (within 10 min), it was maintained, and the rats continued running at this constant speed for the remainder of the planned daily exercise duration. During the adaptive training period, rats that failed to adapt to running or sustained fractures were excluded. After completing the fifth day’s training, the rats were weighed. The rats were randomly assigned to the Con, HXL, HXM, HXH, Con + EHS, HXL + EHS, HXM + EHS, and HXH + EHS based on body weight, with 6 rats in each group. Referring to previous studies (Li et al., 2024c), prior to EHS induction, temperature-monitoring capsules (SV223, Flamingo Technology Co., Ltd., Shanghai, China) were implanted in the experimental rats to measure core body temperature (Tc) at 5-min intervals, while gavage control rats underwent sham surgery as a control. The capsule implantation procedure followed our previously established protocol (Li et al., 2021). Briefly, each rat was anesthetized with isoflurane, and a sterilized temperature-monitoring capsule (1.5 cm × 0.5 cm, 2 g) was surgically implanted into the abdominal cavity through a small aseptic incision. The rats were allowed to rest for 2 days post-surgery. On the 8th day, EHS induction was performed by placing the treadmill into an artificial climate chamber (LTH-575N-01, Drawell Scientific Instrument Co., Ltd., Shanghai, China) set to a temperature of 40°C ± 1°C and a relative humidity of 60% ± 5%. The rats exercised under these high-temperature and high-humidity conditions following the aforementioned exercise protocol. Core body temperature and physical condition were continuously monitored in real time. When the rats’ core temperature exceeded 42°C and they exhibited signs of unconsciousness, they were removed from the chamber and placed in a room-temperature environment (He et al., 2022). After 3 h, samples, such as blood serum, plasma, tissue from organs, and fecal matter were collected from the rats. Non-EHS induction rats with the same gavage protocol were processed simultaneously.

### 2.4 Blood sample examination

Serum biochemical indices, including blood urea (BU), creatine kinase (CK), creatinine (CREA), alanine aminotransferase (ALT), and aspartate aminotransferase (AST), were assessed to evaluate metabolic and organ function parameters. Each parameter provides critical insights: BU and CREA are indicative of renal function, CK reflects muscle damage or stress, while ALT and AST serve as markers for liver health and hepatocellular injury. These measurements were performed using a fully automated biochemical analyzer, the HITACHI 7080 (Tokyo, Japan).

### 2.5 Western blot analysis

Intestinal tissues were rinsed with PBS (Biosharp, BL302A, AnHui, China), cut into 1 cm segments, and placed in 1.5 mL centrifuge tubes. The tubes were snap-frozen in liquid nitrogen and stored at −80°C. For protein extraction, the frozen tissues were lysed on ice using RIPA buffer (Beyotime, P0013B, Shanghai, China) with a protease and phosphatase inhibitor cocktail (Roche, 04906837001/5892970001, Germany). After grinding for 5 min and lysing on ice for 30 min, lysates were centrifuged at 12,000 rpm for 10 min, and the supernatants were collected. Protein concentrations were measured using the Omni-Rapid™ kit (Epizyme, ZJ103, Shanghai, China). Equal protein amounts (20 µg) were separated on 4%–12% SurePAGE™ gels (GenScript, M00654, Nanjing, China) and transferred to PVDF membranes (Millipore, ISEQ00010, Germany) with an eBlot™ L1 Fast Transfer System (GenScript, L00686C, Nanjing, China). Membranes were blocked for 1 h at room temperature with Quick Blocking Buffer (Beyotime, P0252, Shanghai, China), then incubated overnight at 4°C with primary antibodies: ZO-1 (Proteintech, 21773-1-AP, 1:5000), E-cadherin (20874-1-AP, 1:20000), Occludin (27260-1-AP, 1:5000), phospho-p38 MAPK (CST, 4511, 1:1000), p38 MAPK (Beyotime, AF7668, 1:1000), phospho-ERK1/2 (CST, 4370, 1:2000), ERK1/2 (Beyotime, AF1051, 1:1000), phospho-JNK1/2/3 (Beyotime, AF1762, 1:1000), JNK1/2/3 (AF1048, 1:1000), phospho-NF-κB p65 (Abclonal, AP1460, 1:1000), NF-κB p65 (CST, 8242, 1:1000), phospho-IκBα (Abclonal, AP0707, 1:1000), IκBα (Beyotime, AF5204, 1:1000), and GAPDH (Proteintech, 60004-1-Ig, 1:50000). After washing with TBST (Epizyme, PS103, Shanghai, China), membranes were incubated with goat anti-rabbit/mouse IgG (HRP) secondary antibody (Abways, AB0101/AB0102, Shanghai, China, 1:8000) for 2 h. Protein bands were visualized using the Omni-ECL™ chemiluminescence kit (Epizyme, SQ201, Shanghai, China) and imaged with an Amersham Imager 600 (GE Healthcare). ImageJ (NIH, v1.52e) was used for quantification. Experiments were performed in triplicate for each group.

### 2.6 Histological examination and transmission electron microscopy

Organ tissue samples were processed following the protocol outlined in our previous study (Li et al., 2024d). Briefly, the specimens were fixed, dehydrated, embedded in paraffin, sectioned, and stained with hematoxylin and eosin (H&E). The prepared H&E slides were examined under a light microscope (Leica DM2000, Wetzlar, Germany) and digitized using a Panoramic MIDI slide scanner (3DHISTECH, Hungary) for detailed analysis. Additionally, paraffin-embedded intestinal sections were used for immunofluorescence staining. The process involved deparaffinization, rehydration, antigen retrieval, circling, blocking of endogenous peroxidase, serum blocking, incubation with the primary antibody E-cadherin (Servicebio, GB12083; 1:2000, mouse), Occludin (Servicebio, GB111401; 1:2000, rabbit), ZO-1 (Servicebio, GB115686; 1:2000, rabbit), incubation with the corresponding HRP-conjugated secondary antibody, addition of TSA-FITC solution, microwave treatment, incubation with a second primary antibody, quenching of spontaneous fluorescence, DAPI counterstaining for nuclei, and mounting. Detection and image collection were performed using a fluorescent microscope (NIKON ECLIPSE TI-SR, Tokyo, Japan), with consistent image acquisition settings maintained throughout the process (NIKON DS-U3). Intestinal samples were prepared for transmission electron microscopy (TEM) by fixing in 1% OsO₄ in 0.1 M PBS (pH 7.4) for 2 h at room temperature. After fixation, the samples were dehydrated, embedded, polymerized, and sectioned into 60–80 nm slices. These sections were stained with 2% uranyl acetate and 2.6% lead citrate, then analyzed using a Hitachi-7000 electron microscope (Naka, Japan).

### 2.7 Gut microbiota sequencing and analysis

Fecal samples were processed following a previously established protocol. DNA was extracted using the E. Z.N.A.^®^ Soil DNA Kit (Omega Bio-tek, Norcross, GA, United States), and PCR amplification was performed with the AxyPrep DNA Gel Extraction Kit (Axygen Biosciences, Union City, CA, United States). Sequencing was conducted on an Illumina MiSeq PE300 platform (San Diego, United States). Raw 16S rRNA sequencing data were analyzed using the QIIME and i-Sanger platforms (Majorbio BioTech Co., Ltd., Shanghai, China) and deposited in the Sequence Read Archive (SRA) under accession PRJNA1186085. Microbial α-diversity was assessed to evaluate community diversity, while β-diversity variations were analyzed using principal coordinate analysis (PCoA) at the OTU level with weighted UniFrac distances. Group differences were evaluated using ANOSIM/Adonis, and the Wilcoxon rank-sum or Mann–Whitney U test was applied for comparisons at the phylum and genus levels. Bar graphs illustrating species composition were generated based on taxonomic and statistical analyses. LEfSe (Linear Discriminant Analysis Effect Size) was used to identify significantly enriched taxa and biologically relevant features between groups. This study investigated the effects of HXZQD on gastrointestinal microbiota.

### 2.8 Statistical analysis

Experimental results are presented as mean ± standard error. Statistical analyses were performed using GraphPad Prism (v10.3.1, GraphPad Software, CA, United States), except for microbiota data, which underwent multivariate and advanced statistical methods as previously described (Li et al., 2024a; Li et al., 2021). Group comparisons were conducted using one-way ANOVA followed by the Tukey–Kramer multiple comparison test. The two-tailed Student’s t-test was applied for comparisons between two independent groups. Statistical significance was defined as *P ≤ 0.05*.

## 3 Results

### 3.1 Metabolites analysis of HXZQD

HXZQD is a modernized formulation based on Huoxiang Zhengqi San, typically extracting core components such as patchouli oil, perilla oil, and tangerine peel (volatile components), and utilizing dripping pill technology for its production. Given the presence of volatile metabolites in HXZQD, we employed a combined analytical approach using UPLC-Q-TOF-MS and GC-MS to comprehensively identify its primary metabolites. As demonstrated by the UPLC-HRMS base peak ion chromatograms (BPI) in both positive and negative ion modes and the UPLC UV chromatogram (UV at 254 nm) (Figures 1B–D), a total of 64 metabolites were identified from HXZQD through UPLC-Q-TOF-MS analysis (Table 1). As shown in the Total Ion Chromatogram (Figure 1E), a total of 24 metabolites were identified from HXZQD through GC-MS analysis, based on comparison with the NIST database (Table 2).

**TABLE 1:** Main chemical metabolites identified from HXZQD using ultra-performance liquid chromatography-quadrupole time-of-flight mass spectrometry.

No.	Rt (min)	Adducts	Measured *m/z*	Expected *m/z*	Mass error ppm	Formula	Metabolite identification	MS/MS fragment ions	CAS
1	0.88	[M + H]^+^	156.1008	156.1019	−7.1	C_8_H_13_NO_2_	Arecaline	—	63-75-2
2	2.30	[M + H]^+^	268.1042	268.1040	−3.8	C_10_H_13_N_5_O_4_	Adenosine	136.0611; 119.0339	58-61-7
3	8.45	[M-H]^-^	385.0788	385.0776	3.0	C_16_H_18_O_11_	2-[(2E)-3-(4-hydroxy-3-methoxyphenyl)-2-propenoate]-D-Glucaric acid	385.0790; 209.0307; 191.0204; 85.0296	121210-29-5
4	8.90	[M-H]^-^	461.1673	461.1665	1.8	C_20_H_30_O_12_	Verbasoside	461.1666; 315.1083; 205.0739; 135.0443	61548-34-3
5	9.26	[M-H]^-^	385.0783	385.0776	1.7	C_16_H_18_O_11_	2-[(2E)-3-(4-hydroxy-3-methoxyphenyl)-2-propenoate]-D-Galactaric acid	385.0812; 209.0312; 191.0208; 134.0374	108043-98-7
6	9.80	[M + FA-H]^-^	493.2319	493.2291	5.8	C_21_H_36_O_10_	Atractyloside A	493.2311; 447.2264; 285.1708; 179.0557	126054-77-1
7	10.10	[M-H]^-^	385.0791	385.0776	3.8	C_16_H_18_O_11_	3-[3-(4-hydroxy-3-methoxyphenyl)-2-propenoate]-Hexaric acid	385.0789; 209.0322; 191.0195; 147.0298	1801723-82-9
8	10.54	[M-H]^-^	385.0810	385.0776	8.7	C_16_H_18_O_11_	2-[3-(4-hydroxy-3-methoxyphenyl)-2-propenoate]-Hexaric acid	209.0315; 191.0207; 147.0298	1801723-81-8
9	10.65	M^+^	314.1750	314.1751	−0.2	C_19_H_24_NO_3_^+^	Magnocurarine	314.1736; 269.1161; 237.0899; 209.0963; 175.0740	6801-40-7
10	10.91	[M-H]^-^	353.0894	353.0878	4.5	C_16_H_18_O_9_	Chlorogenic acid	191.0565; 173.0456; 161.0243; 135.0453	327-97-9
11	10.91	[M + FA-H]^-^	417.1428	417.1402	6.1	C_17_H_24_O_9_	Syringin	354.0928; 209.0840; 191.0568; 179.0354	118-34-3
12	11.60	[M + FA-H]^-^	523.1700	523.1668	6.0	C_20_H_30_O_13_	Magnoloside S	477.1650; 293.0893; 233.0683; 149.0462; 125.0248	1946004-92-7
13	11.95	[M-H]^-^	385.0788	385.0776	3.0	C_16_H_18_O_11_	2-[3-(4-hydroxy-3-methoxyphenyl)-2-propenoate]-Hexaric acid Isomer	385.0823; 209.0315; 191.0213; 129.0196	—
14	12.85	[M + FA-H]^-^	431.1930	431.1917	1.7	C_19_H_30_O_8_	Roseoside	431.1926; 385.1884; 223.1348; 205.1239; 161.0463; 153.0918	54835-70-0
15	12.92	M^+^	342.1690	342.1700	−2.9	C_20_H_24_NO_4_^+^	Magnoflorine	342.1653; 297.1127; 282.0892; 265.0875	2141--09-5
16	13.60	[M-H]^-^	785.2537	785.251	3.5	C_35_H_46_O_20_	Magnoloside B	785.2590; 623.2260; 477.1652; 161.0247	116872-05-0
17	14.13	[M-H]^-^	593.1540	593.1512	4.7	C_27_H_30_O_15_	Vicenin-2	593.1536; 503.1201; 473.1109; 383.0770; 353.0663	23666-13-9
18	14.89	[M + FA-H]^-^	787.2351	787.2302	6.2	C_33_H_42_O_19_	Narirutin-4′-glucoside	741.2293; 579.1744; 433.1150; 271.0617	17257-22-6
19	15.52	[M-H]^-^	623.2018	623.1981	7.5	C_29_H_36_O_15_	Verbascoside	623.2017; 461.1685; 315.1088; 161.0245	61276-17-3
20	17.42	[M-H]^-^	417.1213	417.1191	5.3	C_21_H_22_O_9_	Neoliquiritin	255.0682; 135.0093; 119.0504	5088-75-5
21	17.53	[M-H]^-^	549.1645	549.1614	5.7	C_26_H_30_O_13_	Liguiritigenin-7-O-D-apiosyl-4′-O-D-glucoside	549.1622; 429.1045; 255.0651; 135.0074	19979-12-8
22	17.84	[M-H]^-^	417.1208	417.1191	4.1	C_21_H_22_O_9_	Liquiritin	417.1184; 255.0655; 135.0085; 119.0495	551-15-5
23	18.00	[M-H]^-^	549.1642	549.1614	5.2	C_26_H_30_O_13_	Liquiritin apioside	549.1638; 255.0655; 135.0083	74639-14-8
24	19.19	[M-H]^-^	623.2022	623.1981	6.5	C_29_H_36_O_15_	Magnoloside A	623.2005; 461.1680; 315.1094; 161.0245	113557-95-2
25	20.22	[M-H]^-^	579.1754	579.1719	6.0	C_27_H_32_O_14_	Naringin	579.1794; 271.0631; 151.0042	10236-47-2
26	21.07	[M-H]^-^	623.2012	623.1981	5.1	C_29_H_36_O_15_	Magnoloside D	623.1994; 461.1664; 315.1083; 161.0242	1418309-03-1
27	21.82	[M-H]^-^	609.1863	609.1825	6.2	C_28_H_34_O_15_	Hesperidin	609.1856; 301.0720; 286.0488; 242.0591	520-26-3
28	23.05	[M-H]^-^	651.161	651.1567	6.6	C_29_H_32_O_17_	Limocitrunshin	651.1636; 549.1312; 507.1218; 345.0649; 330.0418	2010112-95-3
29	23.80	[M-H]^-^	549.1634	549.1614	3.7	C_26_H_30_O_13_	Licuraside	549.1654; 297.0765; 255.0659; 135.0085	29913-71-1
30	24.45	[M-H]^-^	417.1206	417.1191	3.6	C_21_H_22_O_9_	Neoisoliquiritin	417.1197; 255.0658; 148.0160; 135.0084	59122-93-9
31	24.53	[M-H]^-^	549.1621	549.1614	5.7	C_26_H_30_O_13_	Isoliquiritin apioside	549.1635; 429.1055; 255.0662; 135.0082	120926-46-7
32	24.87	[M-H]^-^	711.2916	711.2870	6.5	C_34_H_48_O_16_	Nomilinic acid glucoside	711.2882; 651.2682; 607.2773; 275.1663; 161.0457	125107-15-5
33	25.21	[M-H]^-^	417.1210	417.1191	4.5	C_21_H_22_O_9_	Isoliquiritin	255.0633; 135.0093; 119.0506	5041-81-6
34	25.58	[M-H]^-^	255.0673	255.0663	4.0	C_15_H_12_O_4_	Liquiritigenin	255.0650; 135.0078; 119.0493; 91.0178	578-86-9
35	26.26	[M + FA-H]^-^	349.0943	349.0929	4.0	C_16_H_16_O_6_	Oxypeucedanin hydrate	201.0196; 173.0241; 145.0297; 117.0340	2643-85-8
36	27.25	[M + H-H2O]^+^	317.1014	317.1020	−1.8	C_17_H_18_O_7_	Byakangelicin	218.0206; 203.0331; 188.0101; 175.0388; 160.0148	482-25-7
37	27.40	[M + FA-H]^-^	639.1990	639.1931	9.3	C_28_H_34_O_14_	Poncirin	593.9290; 327.0900; 309.0788; 285.0781	14941-08-3
38	31.43	[M-H]^-^	723.2206	723.2142	8.9	C_33_H_40_O_18_	Natsudaidain-3-O-[3-hydroxy-3-methylglutarate (1→6)]-β-glucoside	723.2216; 417.1217; 402.1003; 359.0793; 329.0405	1179358-72-5
39	32.03	[M-H]^-^	983.4561	983.4493	6.9	C_48_H_72_O_21_	Licorice saponin A3	983.4616; 821.4028; 351.0562	118325-22-7
40	33.41	[M-H]^-^	837.3997	837.3914	9.9	C_42_H_62_O_17_	Uralsaponin N	837.4022; 661.3671; 351.0593; 193.0348	1616062-79-3
41	33.74	[M-H]^-^	329.2343	329.2333	2.9	C_18_H_34_O_5_	Tianshic acid	329.2350; 229.1448; 211.1346; 183.1385; 171.1027	292039-79-3
42	34.44	[M-H]^-^	255.0675	255.0663	4.8	C_15_H_12_O_4_	Isoliquiritigenin	255.0669; 135.0085; 119.0500; 91.0186	961-29-5
43	35.62	[M-H]^-^	837.3993	837.3914	9.4	C_42_H_62_O_17_	Licoricesaponin G2	837.1006; 351.0595; 193.0355	118441-84-2
44	37.56	[M-H]^-^	821.4028	821.3965	7.7	C_42_H_62_O_16_	Glycyrrhizic acid	821.4022; 351.0575	1405-86-3
45	39.38	[M-H]^-^	821.4020	821.3965	6.7	C_42_H_62_O_16_	Uralsaponin B	821.3970; 351.0429; 193.0352	105038-43-5
46	39.97	[M-H]^-^	821.4036	821.3965	8.6	C_42_H_62_O_16_	Licoricesaponin K2	821.4025; 351.0571	135815-61-1
47	40.67	[M + H]^+^	433.1464	433.1493	−6.7	C_22_H_24_O_9_	3′,4′,3,5,6,7,8-Heptamethoxyflavone	433.1455; 418.1222; 403.0991; 385.0898; 373.0530; 345.0563	1178-24-1
48	40.75	[M-H]^-^	823.4186	823.4122	7.8	C_42_H_64_O_16_	Licorice saponin J2	823.4150; 351.0572; 193.0356; 113.0242	938042-18-3
49	41.82	[M + H]^+^	373.1274	373.1282	−2.1	C_20_H_20_O_7_	Tangeretin	373.1250; 358.1047; 343.0797; 328.0583; 297.0745	481-53-8
50	42.67	[M-H]^-^	807.4233	807.4172	7.5	C_42_H_64_O_15_	Licoricesaponin B2	807.4200; 745.4156; 631.3886; 351.0549; 193.0360	118536-86-0
51	44.19	[M + H]^+^	403.1373	403.1387	−3.6	C_21_H_22_O_8_	Nobiletin	403.1367; 388.1120; 373.0909; 355.0812; 315.0520	478-01-3
52	44.59	[M + H]^+^	271.0956	271.0965	−3.3	C_16_H_14_O_4_	Imperatorin	203.0341; 175.0395; 147.0445; 131.0495	482-44-0
53	44.76	[M-H]^-^	645.3702	645.3644	9.0	C_36_H_54_O_10_	Glycyrrhetic Acid 3-O-Glucuronide	645.3689; 569.3522; 523.3427; 469.3356	34096-83-8
54	46.26	[M + H]^+^	301.1053	301.1071	−5.8	C_17_H_16_O_5_	Phellopterin	233.0425; 218.0199; 173.0234; 162.0318	2543-94-4
55	46.46	[M-H]^-^	265.1248	265.1234	5.3	C_18_H_18_O_2_	Honokiol	265.1233; 249.0916; 224.0828; 223.0758; 209.0605; 197.0604	35354-74-6
56	47.43	[M + H]^+^	271.0959	271.0965	−2.5	C_16_H_14_O_4_	Isoimperatorin	203.0340; 175.0376; 159.0449; 147.0444; 131.0494	482-45-1
57	48.37	[M + H]^+^	520.3357	520.3398	−4.6	C_26_H_50_NO_7_P	1-Linoleoyl-sn-glycero-3-phosphorylcholine	520.3375; 502.3323; 184.0748; 104.1071	22252-07-9
58	48.44	[M-H]^-^	281.1190	281.1183	2.4	C_18_H_18_O_3_	Obovatol	281.1202; 164.0486; 137.0247	83864-78-2
59	48.70	[M-H]^-^	265.1251	265.1234	6.4	C_18_H_18_O_2_	Magnolol	265.1222; 247.1117; 245.0961; 223.0761	528-43-8
60	49.54	[M + H]^+^	496.3369	496.3398	−5.8	C_24_H_50_NO_7_P	1-Palmitoyl-sn-glycero-3-phosphocholine	496.3360; 478.3262; 313.2683; 184.0732	17364-16-8
61	49.97	[M-H]^-^	483.3154	483.3116	7.9	C_30_H_44_O_5_	Poricoic acid B	483.3142; 409.2794	137551-39-4
62	51.44	[M-H]^-^	497.3304	497.3272	6.3	C_31_H_46_O_5_	Poricoic acid A	497.3319; 423.2953; 381.3414	137551-38-3
63	52.75	[M-H]^-^	469.3355	469.3323	6.8	C_30_H_46_O_4_	Glycyrrhetinic acid	469.3365; 425.3466	471-53-4
64	52.96	[M + H]^+^	483.3459	483.3469	−2.0	C_31_H_46_O_4_	Polyporenic acid C	465.3224; 309.2166; 231.1457	465-18-9

**TABLE 2:** Main chemical metabolites identified from HXZQD using gas chromatography-mass spectrometry.

No.	Rt (min)	Formula	Metabolite identification	CAS	Score
1	5.253	C_10_H_14_O	Perillal	2111-75-3	90.93
2	6.97	C_15_H_14_	Caryophyllene	87-44-5	92.49
3	7.14	C_15_H_14_	alpha-Guaiene	3691-12-1	92.05
4	7.35	C_15_H_14_	Seychellene	20085-93-2	90.43
5	7.771	C_15_H_24_	alpha-Farnesene	502-61-4	90.93
6	8.165	C_15_H_24_	delta-Guaiene	3691-11-0	94.79
7	9.726	C_15_H_24_O	Caryophyllene oxide	1139-30-6	87.02
8	10.792	C_15_H_26_O	8-epi-gama-eudesmol	998244-67-9	90.44
9	11.016	C_15_H_26_O	Elemol	639-99-6	89.01
10	11.43	C_15_H_26_O	beta-Eudesmol	473-15-4	92.92
11	11.491	C_15_H_26_O	alpha-Eudesmol	473-16-5	92.18
12	11.777	C_15_H_26_O	Patchouli alcohol	5986-55-0	97.95
13	12.965	C_15_H_24_O_2_	Curdione	13657-68-6	88.58
14	13.962	C_13_H_10_O	Atractylodin	55290-63-6	84.73
15	14.139	C_15_H_26_O	Eudesmol	51317-08-9	80.5
16	14.899	C_15_H_26_O	(+)-Rosifoliol	63891-61-2	80.78
17	17.201	C_16_H_32_O_2_	Palmitic acid	57--10-3	87.92
18	19.129	C_12_H_8_O_4_	Bergapten	484-20-8	84.49
19	20.982	C_18_H_32_O_2_	Linoleic acid	60-33-3	94.73
20	21.084	C_18_H_34_O_2_	Oleic acid	112-80-1	84.99
21	23.222	C_18_H_18_O_2_	Magnolol	528-43-8	90.89
22	25.768	C_19_H_22_O_2_	Androsta-1,4,6-triene-3,17-dione	633-35-2	75.59
23	27.227	C_18_H_18_O_2_	Equilenin	517-09-9	83.69
24	30.357	C_20_H_24_O_2_	5,8,11,14-eicosatetraynoic acid	1191-85-1	80.16

### 3.2 HXZQD alleviated hyperthermia of EHS in rats

**FIGURE 2:**
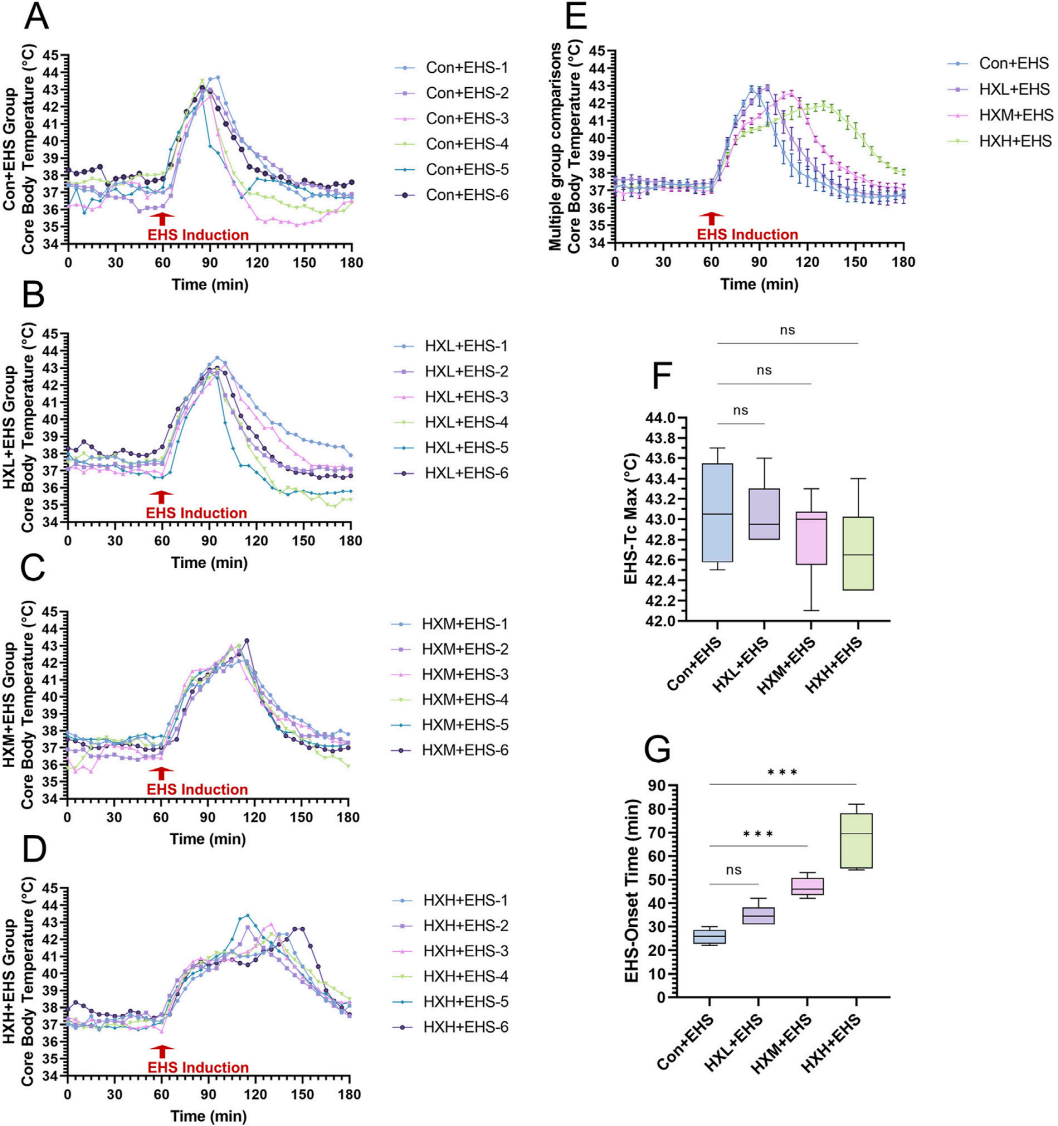
Thermoregulatory response characteristics in EHS rats. **(A–D)** Tc curves of individual EHS rats in four groups during exertional heat stroke induction. **(E)** Tc curves during EHS induction were monitored and compared between four groups. **(F)** Tc Max during EHS induction were compared between four groups. **(G)** EHS onset time were compared between four groups. Data in **(E)** are displayed as the mean ± SEM with six rats per group. Data in **(F, G)** are displayed as a box-and-whisker plot with six rats per group. ^*ns*^ *P* > 0.05*, ^∗∗∗^P* < 0.001.

Compared to classic heat stroke, the rate of Tc increase in rats during EHS induction was faster. Figures 2A–D showed the core temperature curves for each rat in the four groups. Figure 2E presented a comparison of the average Tc across the four groups. A high dose of HXZQD intervention significantly prolonged the rate of Tc increase in EHS rats. We further analyzed the onset time (Figure 2G) and peak Tc (Figure 2F) during the onset period for each EHS rat, and found that the protective effect of HXZQD against EHS showed a dose-dependent response. In summary, a high dose of HXZQD provided the optimal protective effect. As shown in Figure 2F, the peak Tc of EHS rats slightly decreased with increasing concentrations of HXZQD intervention, although the change was not statistically significant. However, medium and high doses of HXZQD intervention significantly prolonged the onset time of EHS in rats, with the differences being statistically significant (Figure 2F).

### 3.3 HXZQD attenuated EHS-induced multiple organ injury

**FIGURE 3:**
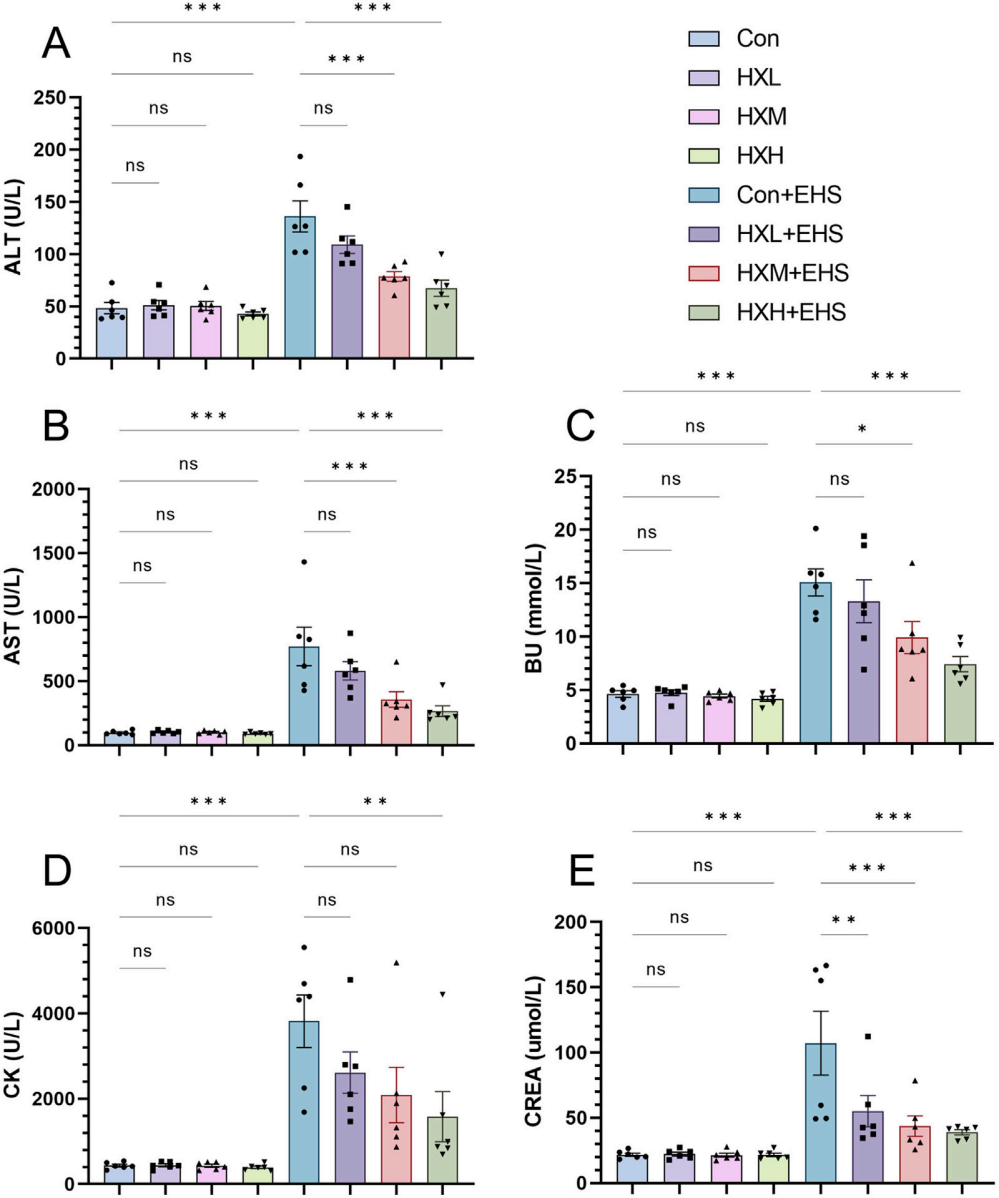
HXZQD decreased levels of blood biochemical markers in rats. The serum biochemical indices ALT **(A)**, AST **(B)**, BU **(C)**, CK **(D)**, and CREA **(E)** were examined by an automatic biochemical analyzer. Data are displayed as means ± SEM, n = 6 per group. ^*ns*^ *P* > 0.05*, ^∗^P* < 0.05, *^∗∗^P* < 0.01*, ^∗∗∗^P* < 0.001.

Organ injury in rats was assessed by monitoring biochemical markers in the blood. ALT, AST, BUN, CK, and CREA are biochemical markers that reflect the functional status of major organs in the body. Analysis of biochemical markers in non-EHS rats revealed that low, medium, and high doses of HXZQD intervention did not cause organ injury, indicating that the selected intervention doses are within a safe range (Figure 3). Further analysis of biochemical markers in EHS rats revealed that HXZQD intervention effectively alleviates heat stress-induced multiple organ dysfunction. Notably, high-dose HXZQD demonstrated statistically significant improvements across all biochemical markers (Figure 3). Based on the analysis of Tc, high-dose HXZQD exhibited the optimal protective effect against EHS and was identified as the research dose for subsequent molecular mechanism exploration.

### 3.4 HXZQD sustained intestinal barrier function in EHS rats

**FIGURE 4:**
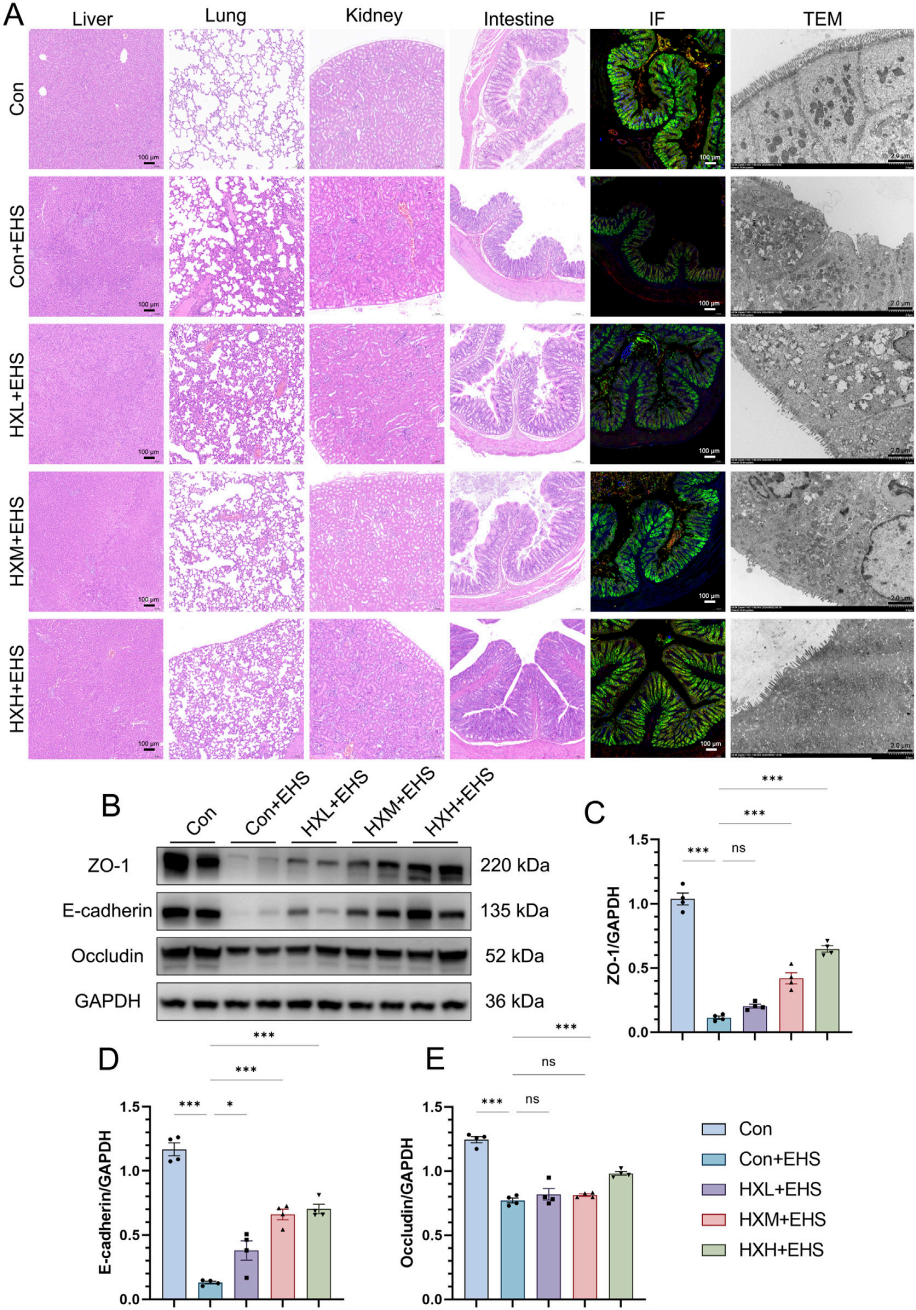
HXZQD sustained intestinal barrier integrity in EHS rats. **(A)** Representative HE images (scale bar, 100 µm) of multiple organs (liver, lung, kidney, and intestine), along with representative immunofluorescence (scale bar, 100 µm) and transmission electron microscopy (scale bar, 2.0 µm) images of the intestine. Cell nuclei were stained using DAPI (blue), and ZO-1 (red), E-cadherin (Green), and Occludin (yellow) proteins were stained with corresponding antibodies. **(B)** ZO-1, E-Cadherin, and Occludin after HS as determined by Western blot examination. GAPDH was used as a loading control. The bar graphs show the band densities of ZO-1 **(C)**, E-Cadherin **(D)**, and Occludin **(E)** relative to that of GAPDH. Data are displayed as means ± SEM, n = 3 per group. ^*ns*^ *P* > 0.05, *^∗^P* < 0.05, *^∗∗^P* < 0.01, *^∗∗∗^P* < 0.001.

The intestinal barrier plays a critical role in the pathogenesis of EHS. Previous studies have also demonstrated that maintaining intestinal barrier function can effectively mitigate the onset of heat stroke (Li et al., 2024b; Li et al., 2021). Our H&E staining results have already demonstrated a significant protective effect of HXZQD in alleviating intestinal injury (Figure 4A). ZO-1, E-cadherin, and Occludin are key proteins essential for maintaining the integrity and function of the intestinal barrier. Using immunofluorescence and Western blot, we examined three representative barrier-associated proteins and found that HXZQD effectively alleviated EHS-induced downregulation of intestinal barrier proteins and disruption of barrier continuity (Figures 4A, B). The protective effect was dose-dependent, with high-dose HXZQD demonstrating a significant protective effect on intestinal barrier function (Figures 4C–E). To further explore the impact of HXZQD on intestinal injury, TEM was performed to examine the ultrastructure of the rat intestines. In line with the histopathological findings, HXZQD preserved the tight junction (TJ) structure and mitigated the disruption of intestinal TJs caused by EHS (Figure 4A). In the Con + EHS group, the intestinal TJ structure was almost completely disrupted, and significant villus loss was observed. In contrast, the HXH + EHS group showed substantial preservation of both intestinal TJ structure and villi, with a marked difference between the groups.

### 3.5 HXZQD regulated the MAPK and NFκB pathways in the intestinal tissues of EHS rats

**FIGURE 5:**
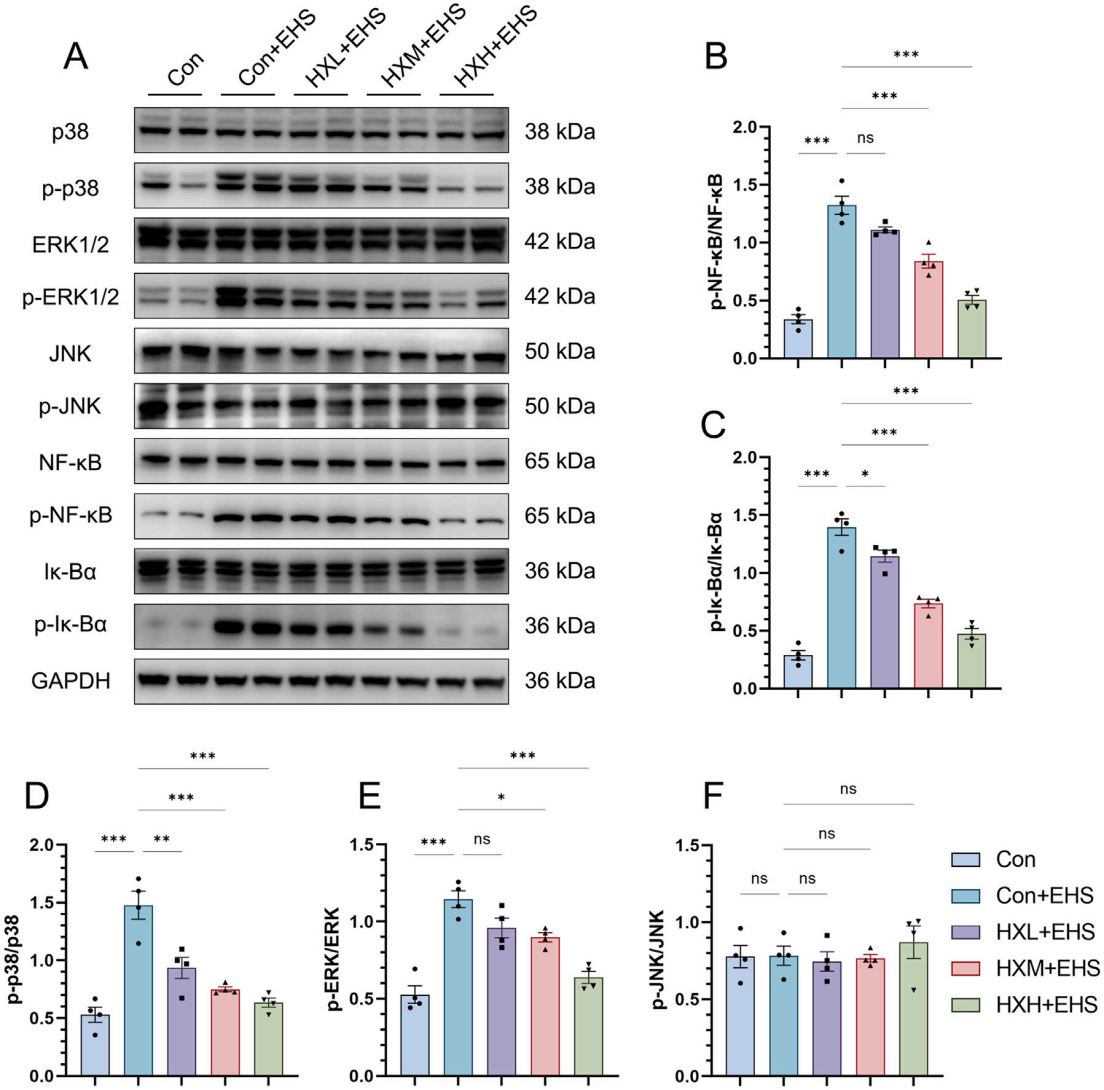
HXZQD regulated the MAPK and NFκB pathways in the intestinal tissues of EHS rats. **(A)** Representative immunoblots of MAPK and NFκB signaling pathway. **(B–F)** The ratios of phosphorylated protein to total protein expression for p38, ERK, JNK, Iκ-Bα, and NFκB. Data are displayed as means ± SEM, n = 3 per group. ^*ns*^ *P* > 0.05, *^∗^P* < 0.05, *^∗∗^P* < 0.01, *^∗∗∗^P* < 0.001.

The mitogen-activated protein kinase (MAPK) and nuclear factor kappa B (NF-κB) pathways play pivotal roles in the response to heat stress and heat stroke. The MAPK pathway regulates stress responses, inflammation, and apoptosis through key components like p38, JNK, and ERK, while the NF-κB pathway controls inflammation, oxidative stress, and cytoprotective mechanisms. Both pathways are closely interconnected, with MAPK enhancing NF-κB activation, leading to amplified inflammatory and stress responses. To investigate the molecular mechanisms involved, we performed Western blot analysis to assess the expression levels of total and phosphorylated proteins for p38, extracellular signal-regulated kinase (ERK), c-Jun N-terminal kinase (JNK), inhibitor of κB alpha (IκBα), and nuclear factor kappa B (NF-κB). Our study found that the phosphorylation levels of p38 and ERK proteins in the MAPK pathway were significantly elevated in the Con + EHS group of rats (Figures 5A, D, E). However, the phosphorylation of the JNK pathway showed no significant changes (Figure 5F). Similarly, the phosphorylation of NF-κB and IκBα proteins was also significantly elevated in the Con + EHS group (Figures 5A–C). HXZQD intervention significantly inhibited the phosphorylation of the aforementioned proteins, including p38, ERK, NF-κB, and IκBα. Notably, the high-dose group (HXH + EHS) demonstrated a statistically significant inhibitory effect on the phosphorylation of proteins associated with both the MAPK and NF-κB pathways. These results were consistent with the high-dose HXZQD intervention demonstrating the most effective outcomes.

### 3.6 HXZQD modulated the structure and composition of the gut microbiota

**FIGURE 6:**
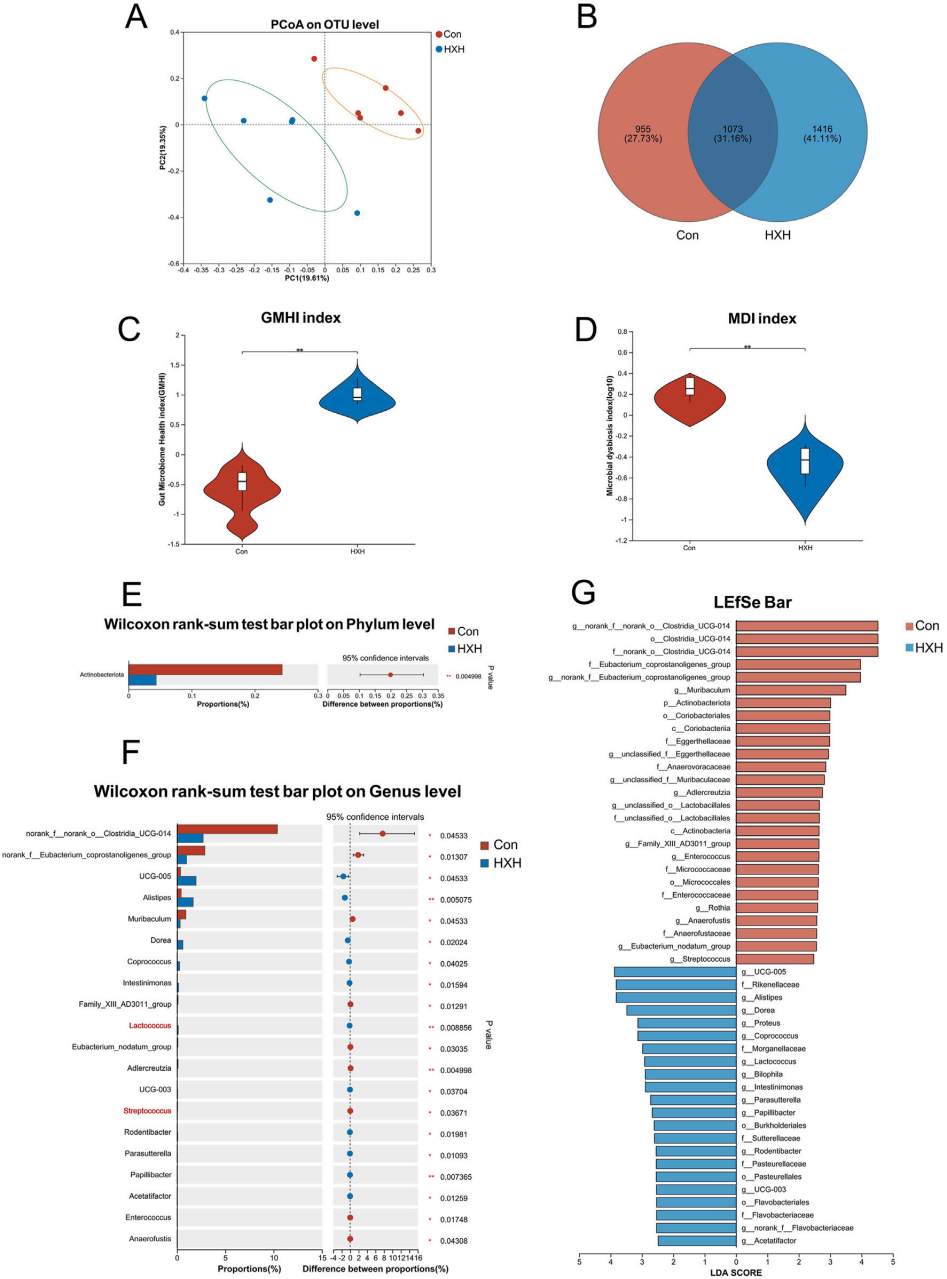
HXZQD modulated the structure and composition of the gut microbiota. **(A)** The β-diversity was calculated by principal coordinate analysis (PCoA). **(B)** A Venn diagram at the OTU level showed shared and unique OTUs between the two groups. **(C, D)** Gut microbiome health index and microbial dysbiosis index were compared between two groups. **(E)** One phylum was found with significant difference between two groups. **(F)** The top 20 abundant genera with significant differences were shown between two groups. **(G)** The gut microbiota composition of the two groups, from the phylum to genus levels, was compared using the linear discriminant analysis effect size (LEfSe) method. The analysis identified bacterial taxa that were differentially abundant between the groups, and their respective linear discriminant analysis (LDA) scores were calculated. Data are displayed as the mean by bar plot analysis. n = 6 per group. *P < 0.05, **P < 0.01.

Oral administration of TCM compounds often impacts the gut microbiota (Zhou et al., 2024; Li et al., 2024c). Therefore, we evaluated the impact of HXZQD on the gut microbiota using 16S rRNA sequencing. PCoA analysis demonstrated that the HXH group differed significantly from the Con group at the OTU level, based on Bray-Curtis analysis. PCoA analysis, based on Bray-Curtis distances, revealed a significant distinction between the HXH group and the Con group at the OTU level (Figure 6A).

V The Venn diagram at the OTU level highlighted distinct differences between the two groups, with only 1,073 OTUs (31.16%) shared, while 955 OTUs (27.73%) were unique to one group and 1,416 OTUs (41.11%) were exclusive to the other (Figure 6B). The Gut Microbiome Health Index (GMHI) assesses health status based on species-level microbial features, while the Microbial Dysbiosis Index (MDI) measures the degree of microbial ecological imbalance. By analyzing the GMHI and MDI indices of the gut microbiota in the Con and HXH groups, we found that HXH significantly increased GMHI and decreased MDI in rats, with both differences being statistically significant (Figures 6C, D). Gut microbiota diversity and richness are assessed through indices like ACE, Chao, and Sobs for species richness, Simpson and Shannon for diversity (richness and evenness), and Coverage for sampling completeness (Supplementary Figure S1). The gut microbiota compositions at the phylum and genus levels were compared using Wilcoxon rank-sum tests. At the phylum level, only one phylum (*Actinobacteriota*) was identified to differ significantly between the two groups (Figure 6E). At the genus level, the top 15 abundant genera with significant differences are shown in Figure 6F. Among these genera, *Lactococcus* was significantly upregulated, while *Streptococcus* was significantly downregulated in the HXH group. The results of our LEfSe analysis (LDA >2.0, *p* < 0.05) were consistent with the pairwise comparisons detailed above (Figure 6G).

## 4 Discussion

Due to the global issue of extreme climate change, human health is increasingly threatened by extreme heat. Among various heat-related illnesses caused by high temperatures, heatstroke is the most severe. Heatstroke can be classified into classic heat stroke and EHS based on the causative factors and susceptible populations (Li et al., 2024d). CHS predominantly affects young individuals, pregnant women, the elderly, and those with chronic illnesses or compromised immune function, while EHS commonly occurs in healthy young people engaging in intense physical activity during summer, such as military personnel, athletes, firefighters, and construction workers. The Expert Group of Heat Stroke Prevention and Treatment of the Chinese PLA found that EHS frequently occurs in young and middle-aged individuals required to work in high-temperature environments, with unpredictable onset, limited targeted preventive measures, and rapid disease progression that remains challenging to mitigate even with intensive care unit interventions (Liu et al., 2020). Developing preventive measures tailored to these EHS-susceptible populations can fundamentally reduce the incidence and mortality rates of EHS. As mentioned above, TCM has been practiced in China for thousands of years, offering extensive experience in disease management. The core principle of TCM emphasizes that prevention is more important than treatment. Our team has previously reviewed various TCM formulations capable of counteracting heat stroke, among which HXZQD is currently the most widely used preventive preparation in China. HXZQD is composed of ingredients primarily derived from medicinal and edible sources, aligning with the “homology of medicine and food” concept. Due to the presence of volatile compounds in HXZQD, we conducted a combined analysis using UPLC-Q-TOF-MS and GC-MS to identify its major components and support the subsequent exploration and validation of its active ingredients. Components like *Atractylodes Rhizome*, *Pericarpium Citri Reticulatae*, *Magnolia Bark* (processed with ginger), *Poria*, and *Licorice* Extract are widely recognized for their dual use in TCM and food. This formulation highlights its safety, functional food potential, and suitability for daily use (Wang et al., 2025). It is commonly taken orally before physical activity or work during the summer or as an emergency supplement during discomfort in high-temperature conditions, effectively mitigating the progression of heat stroke. Previous studies have demonstrated that HXZQD can alleviate acute intestinal injury caused by heat stroke. However, we have noted that this study was primarily based on a CHS rat model that did not include exercise, leaving room for improvement to better reflect real-world application scenarios. Therefore, our team decided to establish a precise EHS rat model to evaluate the protective effects of HXZQD and explore its underlying mechanisms.

To verify the protective effect of HXZQD on EHS, this study established a rat model of exercise-induced EHS under hot conditions based on previous research (Li et al., 2024a; Li et al., 2024b; Li et al., 2021). We observed that many studies on EHS utilize treadmill exercise interventions, which often require forced movement through electrical stimulation. This approach frequently results in toe injuries and bleeding in rats due to the forced exercise (Chao et al., 2023; Wen et al., 2023). In this study, a running wheel was used for exercise intervention, and adaptive training was conducted prior to modeling to ensure that the rats could sustain continuous exercise during the modeling process, thereby ensuring the stability of the exercise intervention as a factor. Drawing on our research team’s experience with using temperature-monitoring capsules to track Tc, we first monitored and compared the thermoregulatory responses of EHS rats in each group. HXZQD has been shown to effectively reduce the rate and extent of Tc elevation during EHS induction in rats, thereby delaying the onset of EHS symptoms. Moreover, this protective effect exhibited a dose-dependent relationship, as evidenced by the results that high-dose HXZQD intervention demonstrated the most effective protective outcome. Further analysis of blood biochemical indices revealed that control rats under three different intervention dose concentrations did not exhibit organ dysfunction. In contrast, rats subjected to EHS induction showed significant multi-organ damage, which was notably alleviated by HXZQD intervention. The study determined that the high-dose HXZQD would be selected for subsequent mechanism exploration and validation. Studies have found that *Huoxiang Zhengqi* has been demonstrated to alleviate intestinal injury and enhance intestinal barrier function in various conditions (Wang et al., 2024; Wu et al., 2024; Dong et al., 2022). EHS is closely linked to intestinal barrier dysfunction, which plays a pivotal role in its pathogenesis (Dmytriv et al., 2024). Elevated Tc during EHS leads to heat stress and intestinal hypoxia, compromising tight junction proteins like ZO-1 and Occludin, thereby increasing intestinal permeability. This allows endotoxins, such as LPS, and other harmful substances to translocate into the bloodstream, triggering systemic inflammation via TLR4/NF-κB pathways. Additionally, oxidative stress exacerbates barrier damage, while gut microbiota dysbiosis further weakens intestinal integrity, creating a vicious cycle. This interplay exacerbates intestinal damage and amplifies the inflammatory response, emphasizing the critical importance of preserving gut integrity in the prevention and management of EHS. Notably, this study successfully demonstrated that EHS-induced intestinal damage was significantly alleviated by HXZQD, as evidenced by the improved expression and distribution of barrier proteins as well as the ultrastructural observations under TEM, both of which support this conclusion. Under heat stress, excessive activation of the MAPK pathway disrupts key proteins such as ZO-1 and Occludin, leading to increased intestinal permeability (Ghulam Mohyuddin et al., 2022). The NF-κB pathway also plays a critical role in intestinal barrier function by modulating inflammation and influencing the integrity of tight junction proteins, thereby affecting gut permeability (Feng et al., 2024). In intestinal barrier research, the MAPK/NF-κB signaling pathway plays a pivotal role in regulating tight junction proteins essential for maintaining barrier integrity (Feng et al., 2024). Furthermore, numerous protective studies based on *Huoxiang Zhengqi* have demonstrated its ability to inhibit the NF-κB signaling pathway (Wang et al., 2024; Tang et al., 2023; Dong et al., 2022). Building on this research background, we investigated the activation of the MAPK and NF-κB signaling pathways and confirmed that HXZQD effectively inhibits the activation of these damage pathways induced by EHS. In this study, HXZQD was administered via oral gavage, and given that many TCM formulas have been shown to significantly regulate gut microbiota positively, we further analyzed the gut microbiota of the rats. This study found that HXZQD significantly increased the proportion of beneficial bacteria, such as *Lactococcus*, while decreasing the proportion of harmful bacteria, such as *Streptococcus*. HXZQD, by promoting the growth of beneficial bacteria such as *Lactococcus*, can increase GMHI. A higher GMHI reflects an improved gut environment with reduced inflammation, enhanced immunity, and better nutrient absorption. MDI quantifies the diversity of microbial species within the gut, reflecting the richness and evenness of the microbial community. A higher MDI of HXZQD indicates a more diverse and resilient microbiota. The positive regulatory effects of HXZQD on gut microbiota may partially explain the enhanced tolerance of rats to heat stress-induced damage during EHS, aligning with findings from our previous preventive studies (Li et al., 2024c; Li et al., 2021). TCM formulas interact closely with the gut microbiota, influencing health through prebiotic-like effects and microbial modulation. Herbal components in TCM promote beneficial bacteria growth, restore microbial balance, and enhance diversity. Gut microbes metabolize TCM compounds into bioactive metabolites, mediating therapeutic effects such as immune regulation and inflammation reduction. HXZQD supports gut barrier function and alleviates dysbiosis. Understanding the HXZQD -microbiota relationship offers opportunities for personalized medicine, targeted therapies, and a deeper scientific foundation for HXZQD’s efficacy.

This study strongly demonstrates the significant protective effects of HXZQD against EHS. Moreover, compared to the effective dosing observed in classic heat stroke studies, EHS requires a higher dosage to achieve notable protective effects. This suggests that healthy individuals engaging in outdoor exercise may need to take higher doses of HXZQD for more effective prevention of EHS. Given that HXZQD is a modernized TCM formulation already approved for use in China, extensive clinical evidence suggests its significant efficacy in protecting against EHS in humans under high-temperature conditions. In the future, we plan to conduct large-scale randomized controlled trials to obtain higher-level evidence of its protective effects against EHS in humans.

## 5 Conclusion

In conclusion, this study demonstrated that HXZQD maintained intestinal homeostasis under EHS by regulating the MAPK/NF-κB signaling pathways to preserve intestinal barrier function and improving gut microbiota composition.

## Data Availability

The original contributions presented in the study are included in the article/supplementary material, further inquiries can be directed to the corresponding authors. The raw 16S rRNA sequencing data presented in the study are deposited in the Sequence Read Archive repository, accession number PRJNA1186085, available at: https://www.ncbi.nlm.nih.gov/search/all/?term=PRJNA1186085.

## Supplementary material

SUPPLEMENTARY FIGURE S1 HXZQD modulated the gut microbiota according to the α-diversity. The ACE **(A)**, Sobs **(B)**, Simpson **(C)**, Shannon **(D)**, Chao **(E)**, and Coverage **(F)** indices of the α-diversity analysis at the OTU level were calculated and compared between the two groups. Data are displayed as the mean by box-and-whisker plot analysis. n = 6 per group. **P* < 0.05.
