# Supplementary figures and images for: Huoxiang Zhengqi dropping pills alleviate exertional heat stroke–induced multiple organ injury through sustaining intestinal homeostasis via regulating MAPK/NF-κB pathway and gut microbiota in rats
Source: Front Pharmacol. 2025 Jan 7;15:1534713. doi: 10.3389/fphar.2024.1534713 (PMC11747358; doi:10.3389/fphar.2024.1534713)

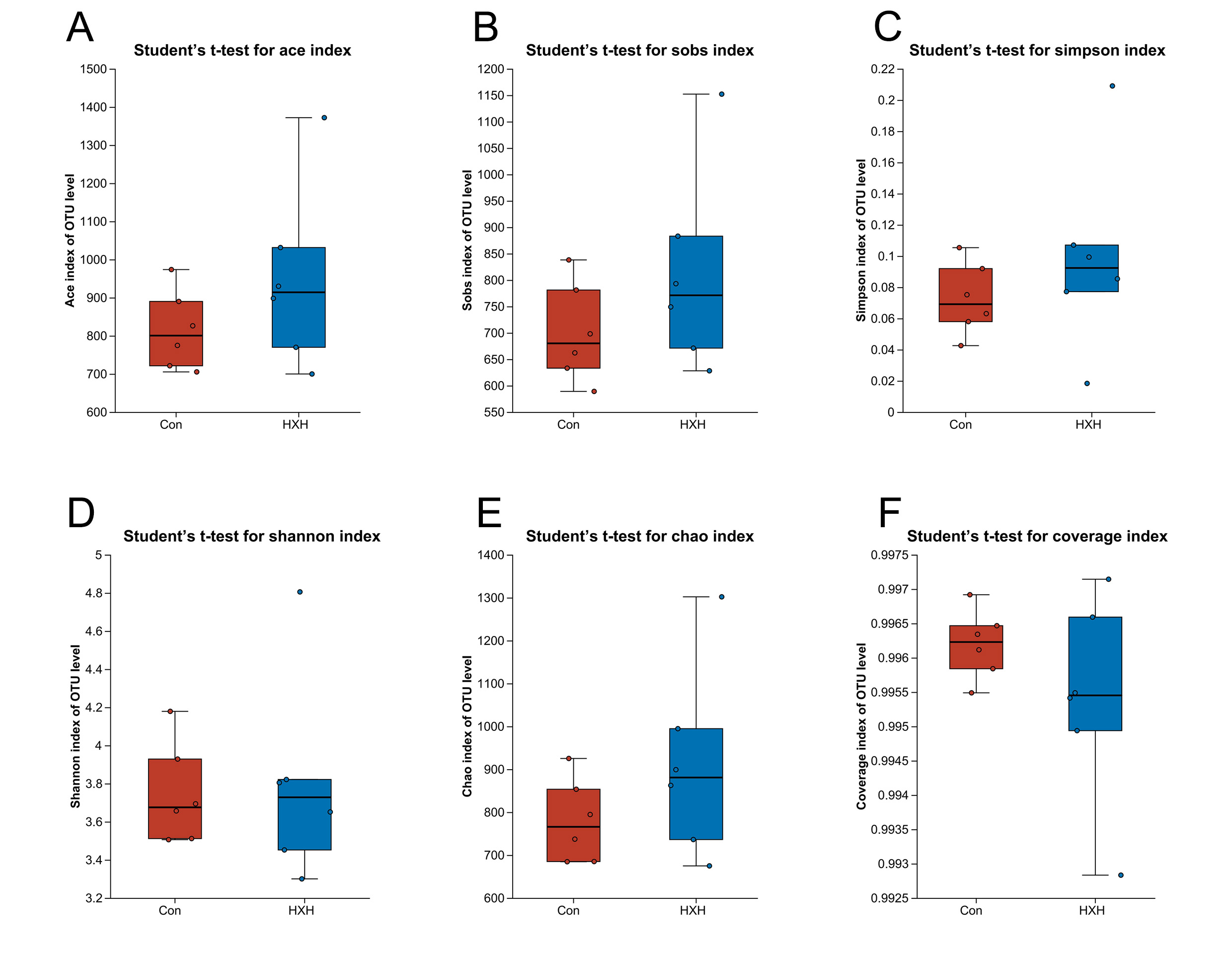

Supplement: Supplementary file 1 [file Image1.jpeg]

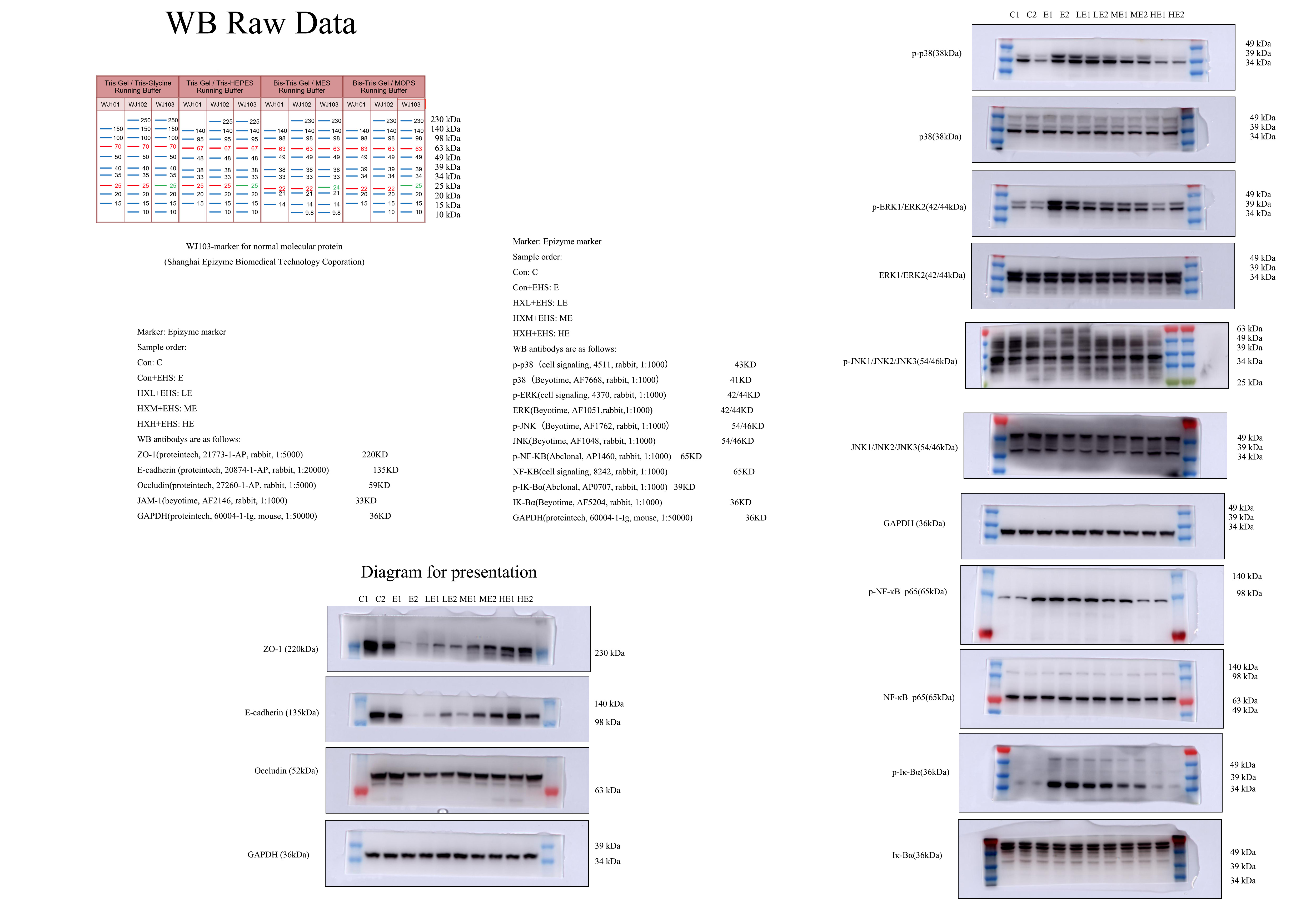

Supplement: Supplementary file 2 [file DataSheet1.zip › Figure4_5-WB raw data.jpg]
